# The Susceptibility to Persuasion Strategies Among Arab Muslims: The Role of Culture and Acculturation

**DOI:** 10.3389/fpsyg.2021.574115

**Published:** 2021-08-12

**Authors:** Momin Alnunu, Azzam Amin, Hisham M. Abu-Rayya

**Affiliations:** ^1^Doha Institute for Graduate Studies, Doha, Qatar; ^2^University of Haifa, Haifa, Israel; ^3^School of Psychology and Public Health, College of Science, Health and Engineering, La Trobe University, Melbourne, VIC, Australia

**Keywords:** social influence, acculturation, immigration, cross-cultural differences persuasion, culture and acculturation, Arabic culture, persuasions strategies

## Abstract

This study is set forth to explore whether the susceptibility to persuasion—as articulated by Cialdini’s persuasion strategies—could vary with culture and acculturation. We examined individuals from the Arabic culture and their susceptibility to persuasion, according to the following strategies: reciprocity, commitment, liking, scarcity, consensus, and authority. The study involved 1,315 Arab Muslims between 18 and 60 years old (*Mean* = 34.65, *SD* = 9.16). The respondents were recruited from among residents of the Arab region (*n* = 507), immigrant Arabs in non-Arabic Muslim countries (*n* = 361), immigrant Arabs in East Asian countries (*n* = 85), and immigrant Arabs in Western countries (*n* = 362). Respondents completed an online Qualtrics survey. Controlling for socio-demographic variables (age, gender, income, education, and length of residence), our results indicated that susceptibility to the strategies differed significantly among Arab Muslims in the Arab region, with reciprocity being the highest and authority the lowest prevailing strategies. The same pattern of susceptibility emerged among immigrant Arab Muslims, regardless of their host country and the acculturation mode (integration, assimilation, separation, and marginalization) they endorse. These findings suggest that there is a consistent persuasion susceptibility pattern in the Arabic Muslim culture that does not seem to be modified by immigration and acculturation modes. Our findings are contrasted with the scarce research on cross-cultural differences in susceptibility to Cialdini’s persuasion strategies.

## Introduction

Persuasion, which has a long-standing in social psychology, denotes a process that targets a desirable change in one’s behavior, attitude, and thoughts (Smith and Mackie, 2015). Correspondently, persuasion strategies are defined as the techniques and mechanisms that can be implemented to change behavior, attitudes, and thoughts (Fogg, 2002; Oinas-Kukkonen and Harjumaa, 2008). Over the past two decades, a number of strategies have been suggested to cause people to perform the desired behavior. Among these, the strategies articulated by Cialdini (2007) have received particular attention in the literature and have become widely used.

Six persuasion strategies, acting as the guide of “Social Influence” and underlying most persuasion attempts were defined, namely reciprocity, commitment, liking, scarcity, consensus, and authority (Cialdini, 2007). To take these in turn, *Reciprocity* means that people generally feel obliged to reciprocate favors, aid, and gifts. In persuasive communication, the reciprocity tendency makes the recipient more susceptible to the influence exerted by the granter of the favor; *Commitment* is based on Festinger’s (1957) Cognitive Dissonance Theory that people strive for internal consistency and dissonance avoidance. Once people commit to doing something, it becomes easier to persuade them to do it since they desire internal self-consistency; *Liking* means that people tend to be influenced by others who they happen to like and accept. They also tend to be influenced by others who share with them important traits such as physical attractiveness and similar values (Burger et al., 2004; Smith and Mackie, 2015); *Scarcity* rests on the idea that people generally tend to assign more value to things that are considered “scarce,” and hence they show a clear desire to possessing and preserving those items; *Consensus* is derived from the Social Conformity Theory (Asch,1951a,b) and means that people generally tend to conform and comply to the values, opinions, and attitudes of others, and they simply do that by seeking peer acceptance or security, especially in ambiguous conditions (Cialdini, 2007; Smith and Mackie, 2015); and lastly, *Authority* indicates that people have a higher likelihood to be influenced by someone who is perceived as an authority due to, for instance, the knowledge, power, or wisdom they possess (Dillard and Shen, 2013).

These persuasion strategies are powerful due to their peripheral influence rather than a central processing of the persuasive message (Cialdini, 2007). The peripheral route allows the message receiver to depend on heuristics, intuition, and emotions while processing, rather than depending on logic, analysis, and facts that characterize the central route to persuasion (e.g., Petty and Cacioppo, 1986; Chaiken et al., 1989). Cialdini (2007) claims that these defined strategies are universal— people of different cultural backgrounds would similarly be susceptible to them.

While the present study welcomes the specification of the six persuasion strategies, it builds on the proposition that their comparative effectiveness may vary across cultures. Culture shapes a wide range of fundamental emotional, behavioral, cognitive, developmental, and social outcomes (Heine, 2015). To wit, cultural differences in susceptibility to persuasion, generally speaking, have been shown to differ by culture (e.g., Pornpitakpan and Francis, 2000; Wosinska et al., 2000; Schouten, 2008; Rodrigues et al., 2018), and cultural differences in susceptibility to Cialdini’s six persuasion strategies, more specifically, have already been highlighted between adults from collectivistic and Western individualistic cultures (e.g., Orji, 2016). Research has reported that reciprocity, commitment, and liking have the highest likelihood of persuading people from both individualistic (North American) and collectivistic (East Asian) cultures. However, East Asians showed higher susceptibility to reciprocity, liking, consensus, and authority, compared to North Americans (Orji, 2016).

One possible interpretation of these cultural differences, we surmise, may relate to independent vs. interdependent self-construal, which, respectively, corresponds to the individualistic vs. collectivistic shaping of the self (Markus and Kitayama, 2010). As Markus and Kitayama have proposed, Western cultural socialization emphasizes an independent self-view, and thus Western members would strive for self-expression, uniqueness, and self-actualization, and their actions are based on personal thoughts, emotions, and goals. On contrast, collectivistic cultures socialize their members for interdependence, emphasizing close connection to others and social harmony. Thus, people with interdependent self-construal would strive to socially fit in and their actions are likely to be based on socially defined norms and expectations. We suggest that East Asians score higher on susceptibility to reciprocity, liking, consensus, and authority (Orji, 2016), since these persuasion strategies focus on fundamental definers of interdependence. Relatedly, persuasion messages that utilize these strategies may cause a priming to interdependence among individuals from collectivistic cultures, and thus cause them to be more receptive.

Our study, to the best of our knowledge, is the first to contribute to the literature by studying the susceptibility to persuasion of adults form the Arabic Muslim culture. Existing research had analyzed the unique characteristics of Arabic persuasion and the role the Arabic language plays in establishing persuasion norms among Arab people. In this regard, Suchan (2014) identified that Arabic persuasion norms are characterized by repetition and paraphrasing, highly ornate language, and strong emotions. Other than this linguistic analysis and that of others (e.g., Anderson, 1989; Aggarwal, 2019) addressing the nature of Arabic persuasion from a linguistic perspective, no psychological research to date had looked at how Arab Muslims respond to persuasion strategies, especially those of Cialdini (2007). The present study was designed to fill in this gap. The Arabic Muslim culture is categorized as collectivistic in the cultural values literature (e.g., Schwartz, 1992, 2006; Hofstede, 2001). However, the Arabic Muslim culture should not be treated as identical to other collectivistic cultures (e.g., East Asian) that have been studied in cross-cultural research in the context of persuasion and social influence susceptibility. As noted by Vignoles et al. (2016), the empirical focus of cross-cultural psychology on contrasting North American samples, as exemplars of individualistic cultures, and East Asians, as exemplars of collectivistic cultures, has resonated with two faults: first, promoting North American samples as prototypical individualism and East Asians as prototypical collectivism, and second marginalizing other world cultures within the scientific cross-cultural psychological research. Rectification of these faults requires investigating a range of cultures classified as individualistic or collectivistic. Research indicates that certain differences in susceptibility to persuasion exist between cultures classified as individualistic.

For instance, Hornikx and Hoeken (2007) studied participants from France and The Netherlands, which are categorized as individualistic cultures in both Hofstede’s and Schwartz’s cultural value models. They reported that Dutch participants were more susceptible to persuasion messages incorporating statistical and expert (authority) evidence, compared to their French counterparts. The authors initially hypothesized that the power distance value, on which France scores higher than the Netherlands, would render French people more susceptible to expert (authority) evidence. Their reported findings went clearly contrary to their hypothesis, and they call for further research into the cultural differences by attending to other cultural values than power distance, which fails to explain the differences between the two studied individualistic groups.

Thus, when two cultures share a particular higher level value characteristic, their value-associated cultural behavioral outcomes should not be presumed unequivocally similar. By studying the susceptibility of Arab Muslims to Cialdini’s persuasion strategies, we hope to shed light on the dissimilarities from the prevailing susceptibility pattern among East Asians who were studied as representatives of collectivistic cultures, enriching, in turn, the cross-cultural psychological literature in this area of inquiry. Although East Asian and Arabic cultures are interdependent cultures that emphasize interpersonal connectedness, the nature of this connectedness can probably be differentiated by the distinctive ‘cultural logics’ of face and honor (Leung and Cohen, 2011), which might also carry consequences to cultural differences in susceptibility to persuasion strategies. While face cultures are more hierarchical and place greater emphasis on in-group harmony and modesty, honor cultures put greater emphasis on the virtue and honor of oneself and one’s group (Leung and Cohen, 2011). Mediterranean cultures, such as the Arabic culture, exemplify honor (Smith et al., 2017). Güngör et al. (2014) compared Japanese (face culture) and Turks (honor Muslim culture) and found that, consistent with the regard for face, Japanese interdependence associates more with conformity and fitting in, whereas Turkish interdependence, consistent with the concern for values of honor, associates more with relatedness and sticking together. Building on this scarce evidence and the propinquity of the Turkish culture to the Arabic Muslim culture, we envisage that Arabs would be more susceptible to persuasion strategies that emphasize connectedness such as reciprocity and commitment and less susceptible to strategies that emphasize authority and consensus. In addition to studying Arab Muslims in their own native cultural context, we propose that immigration and acculturation of people play a role in their susceptibility to persuasion. Acculturation is a process that requires immigrants to adapt to the cultural modes and mindset of the host society (Berry et al., 2011). Given that culture is a factor affecting an individual’s persuasion susceptibility (e.g., Hornikx and Hoeken, 2007; Schouten, 2008; Orji, 2016), acculturation may define an important process that results in the modification of one’s persuasion susceptibility. Acculturation would induce a transformation in the individual’s cultural features that may result in behavioral and cognitive patterns that are no longer identical to the ones prior to acculturation.

The behavioral outcomes of this process can be modified by the cultural distance between the home and host societies (e.g., Kashima and Abu-Rayya, 2014)—the greater the distance, the more challenging the acculturation and its consequent behavioral outcomes. Berry’s (1990) four acculturation modes are very well-known and widely studied within the context of immigrants’ psychological and socio-cultural adaptation (e.g., Berry, 2017; Abu-Rayya et al., 2018; Buchanan et al., 2018). While acculturation studies on Arab Muslim immigrants generated mixed-results regarding their acculturation preferences, with some indicating tendencies toward separation (e.g., Bagasra and Mackinem, 2018) and others indicating a tendency toward an integration mode of acculturation (e.g., Britto and Amer, 2007; Abu-Rayya et al., 2018), integration has generally been associated with higher degrees of adjustment (e.g., Berry, 2017; Abu-Rayya et al., 2018). Yet, no study has explored the relationships between the acculturation modes and persuasion susceptibility among Arab Muslim immigrants, or among immigrants more generally. Therefore, the present study was set forth to also examine the susceptibility to persuasion among acculturating Arab Muslim immigrants in culturally various contexts, using Berry’s modes as a guide.

As conceptualized by Berry (1990), *Integration* denotes those immigrants inclined to maintain their culture of origin and simultaneously adopt important features of the host culture; *Assimilation* defines those immigrants who adopt the host culture and show little interest in maintaining their culture of origin; *Separation* refers to those immigrants who maintain their culture of origin, while having no interest in adapting to the host culture; and *Marginalization* defines those immigrants who show no orientation to either their culture of origin or the host culture. In our perception, possible hypothetical relationships between acculturation and susceptibility to persuasion can be explained by the implementation of the self-construal theoretical perspective. Acculturation can modify immigrant individuals’ self-construal (interdependence vs. independence) (Heine, 2015), and this would adjust their susceptibility to persuasion. For instance, immigrants moving from an interdependent self-construal culture to a host culture that encourages an independent self-view may start perceiving themselves as independent beings and, consequently, converge to a similar pattern of susceptibility to persuasion that prevails among members of the independent self-construal host society. This pattern would be clear among immigrant who endorse the assimilation mode of acculturation, and to some degree also those who endorse integration.

To better understand and articulate the characteristics of the Arabic Muslim culture in persuasion susceptibility terms, we study and contrast four Arab Muslims groups. Specifically, Arab Muslims who are residents of the Arab (mainly Muslim) region, immigrant Arab Muslims acculturating to non-Arabic Muslim countries, immigrant Arab Muslims acculturating to non-Muslim East Asian countries, and immigrant Arab Muslims acculturating to Western societies. Non-Arab Muslim countries share many similar cultural and religious features with the Arab world, thus deemed most culturally close; non-Muslim East Asian countries share with the Arab world certain collectivistic cultural values, but the religious foundations of these counties are far distinct from those in the Arab world, thus their cultural distance from the Arab world was deemed intermediate; and Western societies are most distinct from the Arab world in both cultural and religious foundations.

Given the paucity of research in this area, our study is exploratory in nature and it attempts to:

(1) Investigate the susceptibility of Arab Muslims in the Arab region to Cialdini’s (2007) six persuasion strategies;
(2) Explore how this susceptibility compares to the susceptibility of immigrant Arab Muslims hosted in culturally close or distant cultures; and
(3) Examine whether the acculturation modes endorsed by immigrant Arab Muslims play a role in their susceptibility to the persuasion strategies.

Islamic beliefs, values, and institutions have had a lasting historical influence in shaping the Arabic culture (Frangieh, 2018). As noted by Wekhian (2016), Islam plays a prominent role among Arab Muslims, influencing a wide range of aspects of their cultural traditions and personal lives, even as immigrants. In line with this, Naber (2005) found that Arab Muslims in the Unites States assert their Muslim identity first as their collective identity followed by their Arabic identity. Likewise, Abu-Rayya et al. (2018) found a hierarchical pattern of identification among Arab Muslims in Australia with attachment to their Muslim identity comes in the first place, followed by attachment to their heritage Arabic culture in the second place compared to being Australian. Nonetheless, Arabic Muslim identity should not be confused with religiosity. Research indicates a weak association between religiosity and cultural identification among Arab Muslims (Abu-Rayya and Abu-Rayya, 2009; Abu-Rayya et al., 2018). Further, evidence suggests that attachment to Muslim identity among Muslim immigrants seems integral in promoting their successful acculturation to their host society (Karam, 2020), while religious extremism is a potential barrier hindering their integration into the host society (Wekhian, 2016). Since our study is not intended at religious Muslims, we employ the term ‘Arabic Muslim’ to characterize our respondents. Regardless of factors affected their endorsement of a particular acculturation style, the study is oriented to explore the roles acculturation plays in susceptibility to persuasion.

Previous empirical evidence has shown that individuals differ in their susceptibility to persuasive appeals based on personal-level factors such as gender, age, and education (e.g., Alkış and Temizel, 2015; Orji et al., 2015; Oyibo et al., 2017). Since the present study examines just the Arabic Muslim culture’s role in susceptibility to persuasion, personal-level factors were included as covariates in all analyses pertaining to the study’s aims.

## Materials and Methods

### Participants

A total of 1,315 Arab Muslim adults completed our survey, 507 of whom were residing in the Arab Muslim region (e.g., Jordan, Egypt, Palestine, Algeria, Lebanon, Morocco, Qatar, and Saudi Arabia), 361 were living in non-Arabic Muslim countries (Turkey and Malaysia), 85 were residents of collectivistic c (e.g., China and Japan), and 362 were residents of individualistic Western countries (United Kingdom, France, Canada, Germany, United States, and Australia). Males composed 58.8% of the total sample, and the participant’s age varied from 18 to 60 years old (*Mean* = 34.65, *SD* = 9.16). Overall, participants were fairly well educated, with the majority (92.8%) having completed either a post-secondary diploma (9%), bachelor’s degree (44.6%), or graduate degree and above (39.2%). A bit less than 37% of the respondents indicated their income level to be good or very good; the rest had an average (51.2%) or below average (12.2%) income. There were statistically significant differences between respondents from the four regions in age [*F*(3,1289) = 24.54, *p* < 0.001], and the distribution of gender [χ^2^_(__3__)_ = 35.65, *p* < 0.001], length of residence [χ^2^_(__9__)_ = 621.69, *p* < 0.001], education [χ^2^_(__6__)_ = 101.24, *p* < 0.001], and income [χ^2^_(__6__)_ = 44.66, *p* < 0.001]. Table 1 displays the detailed socio-demographic characteristics of the study respondents in each region of residence. As the figures in Table 1 indicate, Arab Muslim residents of individualistic Western countries tended to be older than those from the other regions, there were notably more male residents of non-Arabic Muslim countries and East Asian countries, and those residents of East Asian countries tended to be more in the graduate degree and above educational category.

**TABLE 1:** Socio-demographic characteristics of respondents by region of residence.

	Arab residents of the Arab region (*n* = 507)	Immigrant Arabs in non-Arabic Muslim countries (*n* = 361)	Immigrant Arabs in East Asian countries (*n* = 85)	Immigrant Arabs in Western countries (*n* = 362)
Age mean (*SD*)	34.03 (8.97)	32.71 (8.12)	32.62 (6.44)	38.03 (10.08)
**Gender**				
Female	47.7%	29.1%	32.9%	44.6%
Male	52.3%	70.9%	67.1%	54.4%
**Length of residence**				
≤3 years	9.9%	48.8%	54.1%	46.6%
3–6 years	5.7%	32.4%	20.0%	24.5%
6–10 years	8.3%	15.0%	22.4%	9.6%
>10 years	76.2%	3.9%	3.5%	19.3%
**Education**				
Secondary or less	5.9%	7.5%	0%	10.2%
Post-secondary diploma	6.3%	9.1%	1.2%	14.6%
Bachelor’s degree	56.0%	41.8%	22.4%	36.6%
Graduate degree and above	31.8%	41.6%	67.4%	38.6%
**Income**				
Below average	10.5%	11.4%	5.9%	16.9%
Average	45.2%	61.4%	64.7%	46.3%
Good/very good	44.3%	27.2%	29.4%	36.8%

### Study Procedure

Ethics approval to conduct the study was granted by the Doha Institute for Graduate Studies, Qatar (approval #DI-IRB-2017-S53). We recruited the sample mainly: (1) by positing a call for participation in our study on various worldwide online Arab forums, blogs, internet chat rooms, and LinkedIn and Facebook groups; (2) through snowball sampling, by asking the participants to share the call with their own Arab peers and social networks; and (3) by posting the call for participation on the authors’ international social networks.

We used an online survey to collect the data for this study. Participants’ identities and that of the involved forums were concealed to comply with the assured full confidentiality. Respondents completed an online self-report research questionnaire, which the authors created using Qualtrics. The study questionnaires were administered to all participants in their native language (Arabic). Translation from the source language to Arabic was initially made by the first author, competent in both Arabic and English, followed by a specialized translation review of an expert in both the source and target languages. Quality and accuracy assurance were subsequently finalized by the other two bilingual psychology-expert authors.

Administering the survey in other languages than Arabic was not feasible as we targeted Arab Muslims from a wide range of countries (e.g., France, Germany, Malaysia, Turkey, United Kingdom, United States, etc.,) and Arabic is their common language despite their geographic heterogeneity. Besides, research suggests that using the native language activates the cultural mindset associated with it (e.g., Seo et al., 2016). Considering that this study’s overarching aim was to understand cultural susceptibly to persuasion strategies, administering the study survey in Arabic to our respondents was deemed most appropriate.

### Measures

Participants provided general demographic questions that sought information on their age, gender, country of residence, income, education, and length of residence in their current country. They also responded to the following measures:

#### Persuasion Strategies

We used the Susceptibly to Persuasion Strategies Scale (STPS) developed by Kaptein et al. (2012). The STPS measures the susceptibility toward Cialdini’s six persuasion strategies. It consists of 26 items measured on a 7-point Likert scale (1 = ‘*strongly disagree*’ to 7 = ‘*strongly agree*’). Five items for *Reciprocity* (e.g., “If someone does something for me, I try to do something of similar value to repay the favor”), five items for *Commitment* (e.g., “I try to do everything I have promised to do”), three items for *Liking* (e.g., “The opinions of friends are more important than the opinions of others”), five items for *Scarcity* (e.g., “I would feel good if I was the last person to be able to buy something”), four items for *Consensus* (e.g., “When I am in a new situation I look at others to see what I should do”), and four items for *Authority* (e.g., “I am very inclined to listen to authority figures”). To test that the STPS items represent six distinct strategies in the present study, STPS’s items were submitted to an exploratory factor analysis (principal axis with oblique rotation), retaining eigenvalues greater than 1. The Kaiser–Meyer–Olkin measure of sampling adequacy was 0.86 and Bartlett’s test of sphericity was significant, χ^2^_(__300__)_ = 9993.92, *p* < 0.001; thus data were determined suitable for factor analysis (Dancey and Reidy, 2017; Tabachnick and Fidell, 2019). *Reciprocity*, *Commitment*, *Scarcity*, *Authority*, and *Consensus* emerged as five distinct factors accounting, respectively, for 20.24, 14.92, 6.78, 6.46, and 5.11% of the observed variance. Each set of items loaded onto their respective factor: loadings ranged between 0.62—0.76 for *Reciprocity*, 0.46—0.77 for *Commitment*, 0.41—0.71 for *Scarcity*, 0.52—0.68 for *Authority*, and 0.38—0.64 for *Consensus*. No cross-factor loadings greater than 0.3 were observed. The *Liking* subscale items did not form a distinct factor, on the one hand, and these items did not cleanly load on one of the other factors, on the other hand. We thus opted to drop *Liking* from our further analyses addressing the study aims. As shown in Table 2, all of the five factor subscales we retained demonstrated sound reliability in the present study. In their original study on 217 respondents, Kaptein et al. (2012) reported a Cronbach’s α reliability ranging from 0.60 to 0.81 for the STPS subscales.

**TABLE 2:** Cronbach’s alpha reliability by region of residence.

Subscale	Arab residents of the Arab region	Immigrant Arabs in non-Arabic Muslim countries	Immigrant Arabs in East Asian countries	Immigrant Arabs in Western countries
Reciprocity	0.79	0.84	0.75	0.90
Commitment	0.79	0.77	0.78	0.78
Consensus	0.60	0.60	0.71	0.60
Scarcity	0.73	0.72	0.66	0.77
Authority	0.71	0.68	0.61	0.73
Home country acculturation orientation^a^		0.82	0.79	0.82
Host country acculturation orientation^a^		0.81	0.60	0.78

*^*a*^Acculturation was not measured among participants in the Arab region.*

#### Acculturation

We adopted the Brief Acculturation Orientation Scale (BAOS) (Demes and Geeraert, 2014). The scale consists of eight items measured on a 7-point Likert scale (1 = ‘*strongly disagree*’ to 7 = ‘*strongly agree*’). Half of the items gauge respondents’ orientation toward their home culture (e.g., “In my resident country it is important to me to have friends from my home country”) and four items measure respondents’ orientation toward the host culture (e.g., “In my resident country it is important to me to take part in their traditions”). To determine whether items of the BAOS represent two distinct acculturation dimensions, BAOS’s items were submitted to an exploratory factor analysis (principal axis with oblique rotation), retaining eigenvalues greater than 1. The respondents’ data were suitable for factor analysis as indicated by the Kaiser–Meyer–Olkin measure of sampling adequacy (=0.80) and the statistically significant Bartlett’s test of sphericity, χ^2^_(__28__)_ = 2913.42, *p* < 0.001, (Dancey and Reidy, 2017; Tabachnick and Fidell, 2019). Home country and host country orientations emerged as two distinct factors accounting for 42.88 and 22.92% of the observed variance, respectively. The BAOS’s items loaded onto their respective factor with loadings ranged between 0.62—0.81 for home country orientation and 0.63—0.81 for host country orientation. In the current study, both acculturation orientation scales exhibited acceptable reliabilities, as displayed in Table 2. These reliabilities are comparable with those originally found by Demes and Geeraert (2014). Using a sizable sample of 1,929 sojourners they reported Cronbach’s α = 0.78 for home culture and 0.72 for host culture subscales.

### Data Analyses

Data were coded and analyses using IBM SPSS 25. We first conducted preliminary analyses to inspect correlations between the study variables. As shown in Table 3, *reciprocity* was associated with older age and higher education; *commitment* was associated with older age, more length of residence, and higher education; *consensus* was just associated with older age; *scarcity* was associated with gender, more length of residence, and higher income; and *authority* was associated with more length of residence and higher income. All of these associations between respondents’ socio-demographic variables (age, gender, length of residence, education, and income) and scores on susceptibility to persuasion strategies were weak. Nevertheless, given that susceptibility to persuasion is the main DV in our study, we opted to control for these socio-demographic variables in the analyses we run to address the study aims. In addition, the four subsamples we recruited differed to statistically significant degree in the structure of their socio-demographic characteristics, as explained in the participants section, giving us a further reason for controlling over these socio-demographic characteristics in the analyses. Our results section is organized into three subsections that correspond to the study aims.

**TABLE 3:** Correlations between the study variables.

	1	2	3	4	5	6	7	8	9	10	11
(1) Age	−										
(2) Gender	−0.07*	−									
(3) Length of residence	0.15**	0.07*	−								
(4) Education	0.05	−0.10**	0.03	−							
(5) Income	0.08**	0.11**	0.22**	0.14**	−						
(6) Reciprocity	0.07*	0.05	0.02	0.07*	0.04	−					
(7) Commitment	0.17*	0.02	0.11**	0.10**	0.10**	0.46**	−				
(8) Consensus	−0.07*	0.05	<0.01	–0.03	–0.02	0.06*	0.04	−			
(9) Scarcity	−0.09**	0.16**	0.08**	0.02	0.08**	0.16**	0.07**	0.28**	−		
(10) Authority	–0.02	<0.01	0.10**	0.02	0.10**	–0.03	<0.01	0.35**	0.36**	−	
(11) Home country orientation	–0.01	0.05	<0.01	–0.04	0.08*	0.17**	0.07**	0.21**	0.18**	0.22**	−
(12) Host country orientation	<0.01	<0.01	<0.01	–0.03	0.05	0.13**	0.17**	0.21**	0.09**	0.18**	0.31**

Boldfaced correlations are significant; *p < 0.01, **p < 0.01.

First, we conducted a Repeated-Measure Analysis of Covariance (RM-ANCOVA) on susceptibility to persuasion, controlling for respondents’ socio-demographics, to explore the susceptibly pattern prevailing among Arab residents of the Arab region (*study aim #1*). RM-ANCOVA was selected because the same respondents were asked to indicate their susceptibility to each of the five persuasion strategies that represent distinct conditions of persuasion, and we wanted to find out which persuasion strategy had higher presence (mean score) among the respondents. RM-ANCOVA can be performed (1) to test changes in mean scores over three or more time points (i.e., on longitudinal data) or (2) to test differences in mean scores under three or more different conditions of the within subject factor (i.e., on cross sectional data) (Dancey and Reidy, 2017; Tabachnick and Fidell, 2019). The nature of our data satisfy the second option.

Second, to explore whether the susceptibly to persuasion pattern among Arabs would differ by immigration to a culturally distant or close country (*study aim #2*), a Mixed Between-Within Subject RM-ANCOVA was conducted on respondents’ susceptibility to persuasion scores, controlling for respondents’ socio-demographics. The between subject factor in this analysis was respondents’ culture of residence (Arab region, non-Arabic Muslim host countries, East Asian host countries, and Western host countries). This set of analysis helps determine whether an interaction between the between-subject factor (e.g., culture of residence) and the within subject factor (e.g., scores on the five persuasion strategies) exists or not (Dancey and Reidy, 2017; Tabachnick and Fidell, 2019). The absence of an interaction effect would be taken to mean that the susceptibly pattern prevailing among the study respondents is consistent across their regions of residence.

Next, to examine how susceptibility to persuasion differs by acculturation mode (*study aim #3*), again a Mixed Between-Within Subject RM-ANCOVA was conducted on respondents’ susceptibility to persuasion scores, controlling for respondents’ socio-demographics. Here, the between subject factor was respondents’ acculturation mode (integration, separation, assimilation, and marginalization).

Before conducting the noted tests, we examined their underlying assumptions. In particular, no significant outliers for each of the susceptibility to persuasion strategies were observed, and visual inspection of histograms and Q–Q plots for each of the strategies indicated normal distribution. To test normality of the data we did not rely on Shapiro–Wilk test as with large sample sizes, as in our case, it is known to reject the null hypothesis (of data being normally distributed) incorrectly in most such examples (Dancey and Reidy, 2017; Tabachnick and Fidell, 2019). Our data violated the assumption of sphericity of the susceptibility to persuasion scores. Mauchly’s test of sphericity turned statistically significant: χ^2^_(__9__)_ = 217.41, *p* < 0.001 in the analysis addressing study aim #1; χ^2^_(__9__)_ = 517.36, *p* < 0.001 in the analysis addressing study aim #2; and χ^2^_(__9__)_ = 471.20, *p* < 0.001 in the analysis addressing study aim #3. Hence, to achieve a more valid critical *F*-value, we relied on the application of the Greenhouse–Geisser correction to the *F*-statistics’ degrees of freedom (Dancey and Reidy, 2017; Tabachnick and Fidell, 2019).

## Results

### Study Aim #1: Persuasiveness of the Strategies Among Arab Residents of the Arab Region

RM-ANCOVA results showed a statistically significant difference among the five persuasion strategies, *F*(4,1630) = 14.09, *p* < 0.001, η^2^ = 0.03. As shown in Table 4, a series of *post hoc* pairwise comparisons with Bonferroni correction for multiple comparisons revealed that susceptibility to reciprocity, followed by commitment, was the highest, and to authority was the lowest among those participants. Participants’ susceptibility to the other strategies fell in between their susceptibility to commitment and authority.

**TABLE 4:** Bonferroni adjusted pairwise comparisons between the persuasion strategies among Arab residents of the Arab region.

	Mean	*SD*
Reciprocity	6.30^a^	0.82
Commitment	5.64^b^	1.03
Scarcity	4.28^c^	1.35
Consensus	4.25^d^	1.23
Authority	3.37^e^	1.25

*Values with different superscript letters differed to statistically significant degree, *p* < 0.001.*

### Study Aim #2: Persuasiveness of the Strategies and Cultural Distance

The results of a Mixed Between-Within Subject RM-ANCOVA disclosed a statistically non-significant culture of residence (Arab region, non-Arabic Muslim host countries, East Asian host countries, and Western host countries) × persuasion susceptibility interaction effect, *F*(11,4309) = 1.25, *p* = 0.253, η^2^ = 0.003. This implies consistency of susceptibly to persuasion among the study respondents across these regions. To further determine that indeed the same pattern of susceptibility found among Arab residents in the Arab region (see the results under study aim #1) exists also among Arab Muslim residents of non-Arabic Muslim countries, East Asian host countries, and Western host countries, we performed a series of *post hoc* RM-ANCOVAs on susceptibility to persuasion for each of these three regions followed by pairwise comparisons with Bonferroni correction for multiple comparisons. As shown in Table 5, the results indicated similarity of the pattern of susceptibility to persuasion for all regions. Precisely, susceptibility to reciprocity, followed by commitment, was the highest, and to authority was the lowest, and participants’ susceptibility to the other strategies fell in between their susceptibility to commitment and authority, exactly as we found among Arab residents in the Arab region.

**TABLE 5:** *Post hoc* RM-ANCOVAs and Bonferroni adjusted pairwise comparisons between the persuasion strategies among immigrant Arabs.

	Immigrant Arabs in non-Arabic Muslim countries		Immigrant Arabs in East Asian countries		Immigrant Arabs in Western countries	
	*M*	*SD*	*M*	*SD*	*M*	*SD*
Reciprocity	6.19^a^	0.94	6.53^a^	0.623	6.28^a^	1.03
Commitment	5.53^b^	0.97	5.88^b^	0.86	5.57^b^	1.03
Consensus	4.23^c^	1.15	4.33^c^	1.24	4.14^c^	1.24
Scarcity	4.00^c^	1.30	4.23^c^	1.16	3.99^c^	1.42
Authority	3.18^d^	1.18	3.18^d^	1.16	3.01^d^	1.26
	F*(4,1228) = 7.39	*p* < 0.001	F*(4,1098) = 11.47	*p* < 0.001	F*(4,274) = 6.35	*p* < 0.001

*F-values refer to RM-ANCOVA analyses in each culture of residence; values with different superscript letters in each column differed to statistically significant degree, *p* < 0.001.

### Study Aim #3: Persuasiveness of the Strategies and Acculturation Modes Among Immigrant Arabs

Prior to analyzing the relationships between immigrant Arabs’ acculturation modes and their susceptibility to persuasion strategies, assignment of immigrant Arabs to each acculturation mode was carried out, as common in the acculturation literature (Berry et al., 2011). Those who scored above each acculturation dimension’s midpoint were assigned to the integration mode (51.66%); those who scored above the home culture scale midpoint but below the midpoint of the host culture scale were assigned to the assimilation mode (25%); those who scored above the host culture scale midpoint but below the midpoint of the home culture scale were assigned to the separation mode (9.30%); and those who scored below each acculturation dimension midpoint were assigned to the marginalization mode (14.04%). A Mixed Between-Within Subject RM-ANCOVA with respondents’ acculturation mode (integration, separation, assimilation, and marginalization) as the between subject factor disclosed a statistically non-significant acculturation mode × persuasion susceptibility interaction effect, *F*(11,2208) = 1.78, *p* = 0.085, η^2^ = 0.008. Additionally, as displayed in Table 6, *post hoc* RM-ANCOVAs followed by pairwise comparisons with Bonferroni adjustment for multiple comparisons indicated that the prevailing pattern of susceptibility among immigrant Arabs did not depend on their acculturation mode. Participants’ susceptibility to reciprocity, followed by commitment, was the highest, and to authority was the lowest, regardless of the acculturation mode they endorse. Participants’ susceptibility to the other strategies fell in between their susceptibility to commitment and authority.

**TABLE 6:** *Post hoc* RM-ANCOVAs and Bonferroni adjusted pairwise comparisons between the persuasion strategies based on the acculturation modes.

	Integration		Separation		Assimilation		Marginalization	
	*M*	*SD*	*M*	*SD*	*M*	*SD*	*M*	*SD*
Reciprocity	6.39^a^	0.77	6.43^a^	0.69	6.26^a^	0.92	5.88^a^	1.33
Commitment	5.71^b^	0.86	5.66^b^	0.98	5.66^b^	0.96	5.33^b^	1.27
Consensus	4.41^c^	1.02	4.10^c^	1.13	4.03^c^	1.25	3.57^c^	1.42
Scarcity	4.11^c^	1.33	4.07^c^	1.20	3.88^c^	1.32	3.88^c^	1.48
Authority	3.28^d^	1.21	3.06^d^	1.05	2.97^d^	1.23	2.70^d^	1.30
	F*(4,1153) = 10.77	*p* < 0.001	F*(4,547) = 3.94	*p* = 0.004	F*(4,168) = 4.05	*p* = 0.003	F*(4,261) = 3.26	*p* = 0.01

*F-values refer to RM-ANCOVA analyses in each culture of residence; values with different superscript letters presented in each column differed to statistically significant degree, *p* < 0.001.

## Discussion

### Susceptibility of Arab Muslims to Persuasion Strategies

Employing the persuasion strategies articulated by Cialdini (2007), we found that Arab Muslim residents of the Arab region were most susceptible to reciprocity followed by commitment. These respondents were less susceptible to scarcity and consensus, and the least to authority persuasion strategies. Our results converge with Orji’s (2016) finding that reciprocity and commitment have the highest likelihood of persuading people from both collectivistic (East Asian) and individualistic (North American) cultures. Thus, by studying Arab Muslims, our findings contribute to accumulated evidence buttressing the cross-cultural validity of this persuasion susceptibility. Persuasion techniques that emphasize reciprocity (being the most highly responded to) are likely to modify individuals’ attitudes, thoughts, and behaviors in the targeted subject matter, regardless of culture.

However, we extend the literature by also showing dissimilarities. Orji (2016) reported that collectivistic East Asians respond more to reciprocity, liking, consensus, and authority. Our findings indicate that Arab Muslims respond less to consensus and the least to authority. Therefore, although East Asians and Arab Muslims are classified as collectivistic cultures in both Hofstede’s (2001) and Schwartz’s (2006) cultural value models, they diverge in susceptibility to consensus and authority persuasion strategies, to which Arab Muslims responded the least. Persuasion techniques that particularly emphasize authority are potentially likely to trigger less susceptibility and even, we envisage, to create antagonism among Arab Muslims. Is this not then counter-intuitive given that Arabic cultures score high on Hofstede’s (2001) power distance dimension and that Arabic cultures are mostly authoritarian?

We offer two possible answers. First, in our post-Arab Spring era—starting in 2010 as a series of anti-government uprisings—we believe that authority, in the general sense, among Arab Muslims is becoming associated with oppression, hostility, and frustration and thus, persuasion messages that link their contents to authority are likely to cause a priming and overwhelmingly negative emotions, causing Arabs to be less receptive and even exhibit reactance to this persuasion strategy. Second, building on Güngör et al.’s (2014) findings, we think that interdependence among Arab Muslims is likely less associated with conformity and more with relatedness and thus they show less susceptibility to persuasion strategies that emphasize authority and more susceptibility to persuasion strategies that emphasize connectedness such as reciprocity, as we found.

Moreover, our study deployed the same Susceptibly to Persuasive Strategies Scale (STPS; Kaptein et al., 2012) using the same 7-point Likert scale as Orji (2016). While the following notion was not addressed in the results section, it is still worth noting that a comparison of Orji’s reported East Asian sample’s reciprocity mean of 5.7 (*SD* = 1.11) to the mean of 6.29 (*SD* = 0.82) among our Arab Muslim residents of the Arab region indicates a significant difference between the two cultures [*t*(660) = 7.17, *p* < 0.001]. Thus, while collectivistic East Asians and Arab Muslims show the highest susceptibility to reciprocity, it seems that Arab Muslims respond to this persuasion strategy more than East Asians. The outstandingly highest susceptibility to reciprocity among Arab Muslims could also be attributed to the Arabic culture-specific values and norms such as generosity and giving back, which evolved historically with the culture and are reinforced in Islam, whereby the violation or non-adherence to these values is consider shameful. Future research on the specific cultural norms and values that would explain Arab’s susceptibility to reciprocity and less to authority is warranted.

The aforementioned subtle dissimilarities between Arab Muslims and East Asians that emerged in this niche of human behavior demonstrate that the cross-cultural psychology field would benefit from and be enriched by examining a wider range of collectivistic cultures. The East Asian samples do not tell the full story of collectivistic cultures. This perspective is also rooted in the dissimilarities found in persuasion susceptibility between the two individualistic French and Dutch cultures (Hornikx and Hoeken, 2007). Positive skewness toward North American samples does not depict the full picture of individualistic societies. Thus, more studies involving a wider range of cultures are needed to continue to extricate this field of enquiry from these limitations, as also noted elsewhere (e.g., Rodrigues et al., 2018).

### Immigration, Acculturation, and Susceptibility to Persuasion Among Arab Muslims

The susceptibility to persuasion pattern and order (excluding liking from the analyses) found among Arab Muslim residents of the Arab region also preserved itself among Arab Muslim immigrants in culturally close non-Arabic Muslim countries, culturally far distant Western countries, and culturally intermediately close East Asian countries. Moreover, we found that Berry’s (1990) acculturation modes play no role in this susceptibility pattern and order; whether Arab Muslims endorse an integration, separation, assimilation, or marginalization mode of acculturation, they still showed more susceptibility to reciprocity and commitment, and less susceptibility to scarcity, consensus, and authority persuasion strategies. These findings extend the cross-cultural psychology literature by showing that susceptibility to persuasion among Arab Muslims seems independent on their specific cultural immigration context and acculturation mode. Thus, the same persuasion reciprocity techniques that work with Arab Muslims in the Arab region can work with those who immigrated to different regions. In contrast, authority persuasion techniques are likely to fail consistently across non-immigrant and immigrant Arab Muslims.

The independence between persuasion strategies and the cultural immigration and acculturation context might just be unique to our Arab Muslim sample. Without replication and cross-cultural comparative studies disentangling cultural differences, no confidence in the uniqueness to the Arab Muslim culture can be ascertained here. Testing whether immigrant individuals from multiple cultural groups respond differently to Cialdini’s (2007) persuasion strategies compared to their home country peers remains a research direction that future studies can undertake to conclude the notion.

While the introduction of this paper articulated the theoretical underpinnings for the possible relationships between immigration, acculturation, and susceptibility to persuasion strategies, the null findings in our sample of Arab Muslims should by no means be taken to presume that Arab Muslims’ mindset and behavioral characteristics remain stable regardless of their cultural context. In fact, as the distribution of the acculturation modes among the sample indicates, Arab Muslims tended to endorse most the integration mode of acculturation (about 52% of the sample) followed by assimilation (25%). Both modes depict immigrant individuals who underwent some level of cultural transformation. Our findings add to existing research that supported the tendency among immigrants toward integration (e.g., Abu-Rayya and Sam, 2017; Berry, 2017; Abu-Rayya et al., 2018). The apparent tendency of our sample to prefer the integration mode of acculturation is also in line with a similar pattern found among adult and youth Arab Muslim immigrants (Britto and Amer, 2007; Abu-Rayya et al., 2018).

Extensive research has also documented a wide range of positive psychological (i.e., emotional) and sociocultural outcomes (i.e., competence) associated with integration endorsement (e.g., Berry, 2017; Abu-Rayya et al., 2018). Yet, our null finding of a relationship between integration and persuasion might indicate that integration endorsement among Arab Muslims was either not strong enough to generate the envisaged effects on susceptibility to persuasion strategies or no such effects exist. In other words, integration endorsement might relate to emotional outcomes and some behavioral skills (i.e., cultural competence) but probably not to other behaviors associated with persuasion.

### Limitations

A number of study caveats should be noted. First, our findings are based on a sizable sample of Arab Muslims from different regions. Nonetheless, our sample was neither random nor representative of Arab Muslims in the studied regions, thus generalizability is limited. Second, despite our efforts to recruit a heterogeneous Muslim sample to minimize the effect of self-selection bias (known also as a volunteer bias) on the results, we cannot preclude the occurrence of self-selection bias in the resulting data. For instance, respondents in our study were fairly educated, and thus the study findings may not apply to Arab Muslims with low educational levels. Third, while culture of residence did not play a role in Arabs’ susceptibility to persuasion, still the apparent smaller sample size of immigrant Arabs in East Asian countries (*n* = 85) did not allow testing interaction effects of acculturation (four modes) and culture of residence (three regions) on susceptibility to persuasion. Fourth, this study employed a cross-sectional methodology to test susceptibility to persuasion among Arab Muslims, thus the causality of the reported relationships cannot be ascertained. Testing cultural effects on susceptibility to persuasion among immigrants can, for instance, be better understood in experimental research designs that would manipulate persuasion strategies and observe whether respondents with different acculturation modes respond differently, a route that future research can undertake. Fifth, the present study is also limited by employing just one form of measuring acculturation and susceptibility to persuasion and by the relatively low reliability of some of the scales. Future research in this field would benefit if corroborating evidence to both attitudinal measures could be obtained. This would allow a more rigorous investigation of the relationships between acculturation and persuasion susceptibility.

## Conclusion

In sum, this is the first study to shed light on the susceptibility to Cialdini’s (2007) persuasion strategies among Arab Muslims, taking cultural nativity, immigration, and acculturation into account. Reciprocity emerged as the highest and authority as the lowest persistently prevailing strategies among our sample regardless of respondents’ cultural context and acculturation mode. We believe these findings are important in the context of the scarce literature in this field on Arab Muslims and the paucity of cross-cultural systematic research on immigration, acculturation and susceptibility to persuasion.

## Data Availability Statement

The raw data supporting the conclusions of this article will be made available by the authors, without undue reservation, to any qualified researcher.

## Ethics Statement

The studies involving human participants were reviewed and approved by Doha Institute for Graduate Studies, Qatar (approval #DI-IRB-2017-S53). The patients/participants provided their written informed consent to participate in this study.

## Author Contributions

All authors listed have made a substantial, direct and intellectual contribution to the work, and approved it for publication. All authors contributed equally to this manuscript and were involved equally in all stages of the study: Design, execution, analyses, and writing up.

## Conflict of Interest

The authors declare that the research was conducted in the absence of any commercial or financial relationships that could be construed as a potential conflict of interest.

## Publisher’s Note

All claims expressed in this article are solely those of the authors and do not necessarily represent those of their affiliated organizations, or those of the publisher, the editors and the reviewers. Any product that may be evaluated in this article, or claim that may be made by its manufacturer, is not guaranteed or endorsed by the publisher.
