# Corrigendum: The Susceptibility to Persuasion Strategies Among Arab Muslims: The Role of Culture and Acculturation

**DOI:** 10.3389/fpsyg.2022.900072

**Published:** 2022-04-12

**Authors:** Momin Alnunu, Azzam Amin, Hisham M. Abu-Rayya

**Affiliations:** ^1^Doha Institute for Graduate Studies, Doha, Qatar; ^2^University of Haifa, Haifa, Israel; ^3^School of Psychology and Public Health, College of Science, Health and Engineering, La Trobe University, Melbourne, VIC, Australia

**Keywords:** social influence, acculturation, immigration, cross-cultural differences persuasion, culture and acculturation, Arabic culture, persuasions strategies

In the published article, there was an error in the affiliations of the third author, Hisham M. Abu-Rayya. Instead of affiliations 1 and 2, this author should be affiliated to affiliations 2 and 3, with the following affiliation added as the new affiliation 2: “University of Haifa, Haifa, Israel”. The previous affiliation 2 should then be renumbered as affiliation 3. The corrected affiliations are shown below.

Momin Alnunu^1*^, Azzam Amin^1^ and Hisham M. Abu-Rayya^2, 3^

^1^Doha Institute for Graduate Studies, Doha, Qatar

^2^University of Haifa, Haifa, Israel

^3^School of Psychology and Public Health, College of Science, Health and Engineering, La Trobe University, Melbourne, VIC, Australia

The authors apologize for this error and state that this does not change the scientific conclusions of the article in any way. The original article has been updated.

## Publisher's Note

All claims expressed in this article are solely those of the authors and do not necessarily represent those of their affiliated organizations, or those of the publisher, the editors and the reviewers. Any product that may be evaluated in this article, or claim that may be made by its manufacturer, is not guaranteed or endorsed by the publisher.

